# Efficacy of different multi-layer hermetic bags on the seed quality of the faba bean (*Vicia faba* L.) in outdoor storage condition

**DOI:** 10.1038/s41598-023-47598-4

**Published:** 2023-11-24

**Authors:** Hayam I. A. Elsawy, Mohamed M. El-Kholy, Amany M. Mohamed, Reham M. Kamel

**Affiliations:** 1 Seed Technology Department, Field Crops Research Institute, Agriculture Research Center, Giza, 12619 Egypt; 2 Agricultural Engineering Research Institute, Agricultural Research Center, Giza, 12611 Egypt

**Keywords:** Plant breeding, Biochemistry

## Abstract

Faba bean seeds' sustainability correlates with the initial quality of cultivated seeds. The duration of storage is a significant factor that can affect the quality retention of any crop seed. Additionally, the hermetic bags effectively influence the quality of crop seeds during the storage process. This study evaluated two faba bean cultivars, Nubaria 1 and Giza 716, after various storage periods of 6, 12, 18, and 24 months. Seeds stored in 3 and 7-layer hermetic bags have shown significantly improved bulk temperature after 12, 18, and 24 months (neither more than ≈ 30 °C nor less than ≈ 15 °C). However, the relative humidity (RH%) increased significantly in both bags and cultivars throughout the storage periods, reaching 61, 59.77% and 59.53, 57.53% at the end of the storage period for Nubaria 1 and Giza 716 inside the 3 and 7-layer bags, respectively. The seeds' germination % decreased significantly (20%) after 24 months at the 3-layers compared with 13.12% at the 7-layer bags for Nubaria 1 with superior germination% of Giza 716 under the same conditions where the decrease in the percentage of germination reached 15.56% and 8.86% reduction for both 3 and 7 layers, respectively. The seedling vigor index exhibited the same trend of germination % with better results of the 7-layer bags for both cultivars. The moisture content (MC%) was substantially elevated by 1% at the end of the storage for both cultivars and bags. After 12 months, beans considerably increased color deterioration, with a loss of 43.16 and 53.60% for Giza 716 and Nubaria 1 stored in 3-layer bags, respectively; however, 7-layer bags were always better than 3-layer bags (with a loss of 32.56 and 45.56%). Furthermore, storage in 7-layer hermetic bags triggered a significant decrease (14.94%) in the total phenolic (TPC) after 24 months for Giza 716 without a substantial difference with Nubaria 1. Additionally, the simulation showed that after 18 months and 24 months of storage, the Nubaria 1 seeds packed in the 7-layer hermetic bags produced total tannins (TTC) 39.1% and 42.5%, respectively, more than those packed in the 3-layer hermetic bags. That TTC had a negative correlation with the testa's darkness. Taken together, the faba bean seeds could be stored for a long period in the 7-layer hermetic bags, preserving seed quality and expanding marketing prospects.

## Introduction

Faba bean (*Vicia faba.* L) is a protein-rich legume that may be eaten fresh or dried. This ancient world plant may be found across Africa, Europe, and Western Asia. Bean is a rainfed cold season crop that thrives in locations with sufficient rainfall, allowing it to thrive in various nations. Because of the money it provides to farmers, it has become a considered crop. It's also significant for soil fertility, human nutrition (as an excellent vegetarian protein source), animal feed, and industrial reasons^1^. It is one of the most significant pulse crops in the nation's agricultural system. Compared to other cultivated grain legumes, it leads the pack in terms of total national production^2^.

For instance, human and domestic animals in Egypt eat faba beans, especially where higher-quality protein is scarce. Faba beans play a significant role in our national diets; around 14 g of faba beans are taken daily per capita, accounting for approximately 3 g of protein. Therefore, after harvesting in May and June, faba bean seeds are stored in various ways and under varied circumstances that may lead to a deterioration of seeds^3^*.*

The storage conditions have a significant influence on the sustainability of seed viability. Besides the differentiation in the storage environmental conditions, the storage package strongly affects seed quality. In recent years, the suitability of hermetic storage systems for several crops, such as maize^4,5^, rice^6^, groundnuts^7^, green coffee, and cowpea, has been assessed^8,9^. Hermetic storage has a long history of significance among the different physical strategies for controlling postharvest microorganisms^10^. The hermetic packaging storage method uses suitably sealed structures to generate an in-package environment with a lower concentration of oxygen and a higher concentration of CO_2_. By regulating the respiration rate, the sealed bag preserves the quality of products for an extended duration. The hermetic bags packed products so tightly that the commodity's aerobic respiration depletes the O_2_ level, resulting in insect mortality^11,12^.

The germination tests can compare the quality of various seed lots. Although the germination percentage is the most popular and practical seed viability test, the seedling vigor is more accurate than the germination %. Furthermore, the viability after storing for different periods should be tested by various quality tests such as the electric conductivity of seeds (EC), the acidity value, and different biochemical compositions as the antioxidant components^11^.

In the faba bean, the seed coat color (testa) is one of the most significant aesthetic aspects in selling its seeds for human consumption. The seed testa color of different types of faba bean ranges from white to purple genetically, although the desired color has been characterized as beige, light tan, or buff^13^. Most faba bean types of seed coats are beige or buff when harvested, but depending on storage time and circumstances, they become light brown, dark brown, or virtually black. Consumers equate dark brown testa seeds with seeds and poor cooking and sensory attributes; hence, they are unacceptable in international markets^14^. In various species of beans, oxidation due to ecological O_2_ has been a critical factor responsible for its discoloration^13^. Discoloration in beans may be involved with the oxidation of phenolic compounds, particularly the condensed tannins, the principal and greatest extensively spread group of flavonoids originating in legume seeds^15^.

Total phenolic compounds display solid antioxidant properties through diverse mechanisms, such as reactive oxygen species (ROS), that prevent the deterioration of the tissues^16^. In addition, tannins are phenolic-based secondary metabolites found in different plant organs. Tannins generated in the plant body are antibacterial and antioxidant. As a result, they assist plants in combating various environmental stresses as long-term storage conditions^17^. However, there are some drawbacks to producing tannins in plant tissues; the fundamental disadvantage of tannins as food is that they absorb and bind with various biomolecules in the digestive tract, including proteins, limiting their nutritional availability to humans and animals.

Utilizing the right packaging materials when storing legume grains is essential for reducing postharvest losses. Polyethylene-lined aluminium foil bags were used for faba bean storage and stored in controlled temperature storage rooms^14^. In addition, storage in tin cans without heating the seeds and in tin cans after heating the seeds was used for faba beans^39^. Throughout 12 months of storage, hermetic bags maintained the faba bean germination percentage above 90%^18^.

Storage conditions also influence different morphological and physical characteristics. The seed length, width, penetration, and shear force are affected negatively by increasing the storage period of faba bean seeds^19^. In addition, by increasing the storage period, the crude protein in the whole seed of faba bean significantly reduced from 30 to > 20%, which may be attributed to the activation of the proteolytic enzymes.

Recently, different methods were used to store faba bean seeds that negatively affected the seeds' quality after a long time. However, not much research touched upon the faba bean storage in hermetic bags, especially in outdoor conditions. Therefore, it is necessary to think of an innovative way to store different varieties of faba beans under outdoor storage conditions. Hermetic storage has been a successful option for air tightness (lower oxygen and higher carbon dioxide.) and moisture content control. Thus, this study aims to test and evaluate the quality changes of two different hermetic bags (3–7 layers) for long-term storage (24 months) of two different cultivars of faba beans (Nubaria 1 and Giza 716). The changes in bean quality were tested and evaluated under the following methodology.

## Materials and methods

### Materials

The storage period was extended for 24 months, from Sept. 2020 to Sept 2022, at the Department of Seed Technology, Agricultural Research Centre (ARC), Egypt, under ambient storage conditions. Freshly harvested and uniformly matured faba bean cultivars seeds (Giza 716 and Nubaria 1) were obtained from the legumes research department, Sakha Agricultural Research Station, Agricultural Research Centre (ARC), Ministry of Agriculture and Land Reclamation, Egypt, for the storage experiment. The obtained commercial cultivars were chosen according to their economic importance and productivity; in addition, they were different in size and nutrition content.

### The storage conditions

Nubaria 1 and Giza 716 cultivars of beans were filled and sealed in two types of hermetic bags (three layers of polyethylene 120 μm, and seven layers of polyethylene 150 μm). Each bag had 25 kg of each cultivar's seeds. For each cultivar, the bags were replicated three times from each type. Following that, samples (500 g of seeds) from each bag of the two cultivars were collected for biochemical and quality parameters. The moisture content of both cultivars was between 13.7 and 14.0% for Nubaria 1 and Giza 716, respectively, at the starting storage time. The initial percentages of germination for both cultivars were recorded (100%). Since the faba bean seeds were stored outdoors while the weather temperature and humidity were recorded every six months during the samplings, the ambient conditions for storage varied according to the season. The stored seeds were tested and evaluated every 6 months until the end of the storage period (24 months). Table 1 describes the specifications of the two examined compacted multi-layer plastic films used for the storage process that were produced by a local Egyptian company (Shouman Co., Egypt).

**Table 1:** Mechanical and physical properties of the 3-layer and 7-layer hermetic bags.

Property	Unit	Method	Value (Mean $\pm$ SD)	
			3 layers, 120 μm	7 layers, 150 μm
Average thickness	µm	DIN 53,370	120.7 $\pm$ 1.27	151 $\pm$ 2.00
2 SEGMA thickness tolerance	%		4.7 $\pm$ 0.9	2.1 $\pm$ 0.10
Width	mm	Internal	442 $\pm$ 0.00	442 $\pm$ 0.00
Coefficient of friction				
Out/out	–		0.38 $\pm$ 0.03	0.32 $\pm$ 0.03
IN/IN			0.19 $\pm$ 0.01	0.44 $\pm$ 0.03
NTR/M		–	–	–
Surface tension	Dyn/CM	DNI ISO 8296	38 $\pm$ 0.00	38 $\pm$ 0.00
Tensile strength at break MD	Mpa	ASTM D882	46.6 $\pm$ 4.01	41.0 $\pm$ 1.00
Tensile strength at break TD	Mpa		43.4 $\pm$ 2.45	37.0 $\pm$ 1.00
Tensile strength at yield MD	Mpa		17.3 $\pm$ 2.06	16.5 $\pm$ 0.50
Tensile strength at yield TD	Mpa		21 $\pm$ 0.70	567.5 $\pm$ 7.50
Elongation at break MD	%		531.7 $\pm$ 47.15	562.5 $\pm$ 12.83
Elongation at break TD	%		563.2 $\pm$ 33.68	537.5 $\pm$ 12.50
Elongation at yield MD	%		7.3 $\pm$ 0.55	13.5 $\pm$ 0.50
Elongation at yield TD	%		7.8 $\pm$ 0.51	13.5 $\pm$ 0.50
Oxygen permeability	Cc/m^2^/day		≤ 450	≤ 0.1
Water vapour permeability	g/m^2^/day		≤ 2	≤ 1

*The mean $\pm$ standard deviation (SD) was used to express the data.

### Experiment's method

According to each hermetic bag, either 3-layers or 7-layers for both cultivars, Samples were collected using a simple truck probe from the top, middle and bottom and mixed together (500 g seeds) to get a representative sample every 6 months. The plastic bag experienced punctures at three distinct spots over the length of the bag. Following the probing of the bags, the apertures were then sealed using specialized plastic tape in order to maintain a hermetically sealed system, similarly, for measuring the temperature, O_2_, CO_2_ and relative humidity. These samples were kept at room temperature, sealed securely in plastic bags, and used for qualitative research. Three replicates of each of the two storage bags (3 and 7-layers of hermetic bags) for the two cultivars were used in the experiment. Every six months, samples for quality characteristics for each treatment were taken and measured three times.

### Seed bulk temperature and relative humidity

Seed bulk temperature and relative humidity inside and outside the examined bags were measured using a temperature and RH meter (Lutron, Model MS-7011, United States).

### Carbon dioxide (CO_2_) and oxygen (O_2_) concentration

Concentrations of CO_2_ and O_2_ were monitored every six months at different positions of each bag using O_2_ and CO_2_ Analyser (VIGAS, Model Box-121, France).

### Moisture content of seeds

The seeds' moisture content was determined using the standard ISTA method^20–22^. Ten grams of seeds were placed in an electric air oven at 103 °C for 17 h, then kept in a desecrator at room temperature for 15 min and weighed by a digital balance with an accuracy of 0.001 g.

### Shearing and penetration force

Seed hardness was quantified using a digital hardness meter (SHIMPO FGC-50, Japan). The highest force required to penetrate and shear compress the seeds was also measured^23^.

### Germination parameters

#### Germination percentage

- The pure seed proportions of the samples were used to make the germination test. One hundred fifty seeds were used per sample, and each sample was replicated 3 times with 50 seeds each. The seeds were then placed on plastic germination boards and using the Slant board system (composed of 5 components: the board, germination paper, thin absorbent paper, an exterior container, and a positioning device). Briefly, for making the germination test, as soon as the seeds are on the slant board covered in germination paper, they are covered with a thin layer of absorbent paper to keep them moist. The positioning device held these slant boards at angles between 70° and 80° in the exterior container that contains water, and the germination room was kept at 20 °C for 14 days. The germination % was measured according to the International Seed Testing Association (ISTA)^23^.
- *Seedling length (cm)* by measuring the (radical length + plumule length)
- *Seedling vigor index* was calculated as described by Leist et al*.*^24^ by applying the formula: $({\text{Seedling vigour index }} = {\text{ Average seedling length }}\left( {\left( {{\text{plumule }} + {\text{ radical}}} \right) \, \left( {{\text{cm}}} \right) \, *{\text{ Germination }}\left( \% \right)} \right)$
- *Seedling dry weight (DW)* was measured by keeping 5 seedlings of each variety as a replicate at 70 °C for 72 days, then recording the dry weight in (g).

### Electric conductivity (EC) of seeds

Twenty-five seeds per replicate and three replicates from each bag were weighed and immersed in a conical flask filled with 250 ml of deionized water for 24 h. After 24 h soaking, the electrical conductivity of the solution was determined with a portable EC meter (9 V-1 AmP, Thermo Electron Corporation, USA). Once the readings were recorded, the electrical conductivity values per gram of seeds were calculated, and the results were expressed in μS cm^−1^ g according to the International Seed Testing Association (ISTA)^20^.

### The crude protein

Identified weight of the finely powdered seeds (ca 0.1 g) was digested using a micro Kjeldahl apparatus with (98% H_2_SO_4_) and (30% H_2_O_2_). The crude protein was calculated by multiplying the total Nitrogen by 6.25, according to Sanful and Darko^25^.

### Colour change

The color of the faba bean seeds was measured using a CR-400 colorimeter (Konica Minolta, Japan). The colorimeter produced three parameters in the Hunter Lab scale: L*, chroma, and hue angle for luminance, color intensity, and visible spectrum color. The white plate served as a reference point for adjusting the machine.

### Total phenolic content (TPC)

TPC was measured according to the method described by Sanful and Darko^25^: faba bean sample (1 g) was dispersed in 10 mL of 80% methanol and ultrasonically extracted for 30 min. The supernatant was collected in a 50 mL flask after centrifugation at 3000 g for five minutes with three replications. Evaporation of the supernatant at 40 °C was carried out using an evaporator in a vacuum. The residues were dissolved in 1 mL of methanol and water (60:40) (ultrasonic bath), and then were transferred to a 50 mL flask. The 50 mL flask was washed twice with 1 mL of methanol/water (60:40). Afterwards, 0.3% MeOH–HCl was filled into the 5 mL flask. A 50 μL of Folin–Ciocalteau reagent was added to a 100-mL aliquot of the resulting solution after 2 min of addition to 2% Na_2_CO_3_. The absorbances were then measured 30 min later at 750 nm. The gallic acid was used as a standard for this study and calculated the concentration in gallic acid equivalents (GAE) milligrams per gram of samples.

### Total tannins content (TTC)

The analyses of tannin content in seeds were performed according to the International Pharmacopoeia and AOAC methods^26^ with some modifications. 3 g of seed powder was infused with 250 mL of deionized double distilled water and then filtered through a 0.45 µm sample filter. 25 mL of the distillation was added into 1 L conical flask, and then 25 mL of indigo solution [0.6%] and 750 mL deionized distilled water was added. The solution was titrated with 0.1 N aqueous solution of KMNO_4_ until the blue-colored solution changed to the golden yellow one. The standard solution of indigo carmine was prepared as follows: 6 g indigo carmine was dissolved in 500 mL of deionized distilled water by heating, and after cooling, 50 mL of 98% H_2_SO_4_ was added. The solution was diluted to 1 L with deionized distilled water and filtered through a 0.2 µm membrane filter. The blank test was carried out by titration of 25 mL indigo carmine and 775 mL double distilled water. All samples were analyzed in duplicates. The tannin percent [%] in the samples was calculated as Eq. 1 follows:

1 $${\text{TTC }} = \, \left[ {{\text{V }}{-}{\text{ V}}_{0} } \right] \, 0.00{4157 } \times { 25}0 \, \times { 1}00/{\text{g }} \times { 25}$$

where V is the volume of 0.1 N aqueous solution of KMNO_4_ used in the titration of the sample and V_0_ is the volume of 0.1 N aqueous solution of KMNO_4_ used in the titration of the blank sample as mL; 0.004157 is the tannins equivalent in 1 mL of 0.1 N aqueous solution of KMNO_4_; g is the mass of the sample taken for the analysis as gram and 250 is the volume of the volumetric flask.

### Acidity value

A sample of 5 g faba bean seeds was ground to obtain a fine powder sample before drying in a grinding mill. Acidity was determined according to the mentioned method in^27^ as follows: the fine powder was homogenized in 200 ml of distilled deionized water with shaking for a while. The homogenate was then filtered through a Whatman number 4 filter, and the acidity value of the supernatant was determined in a 25 ml aliquot with 3–5 drops of phenolphthalein titrated with sodium hydroxide to a pH of 8.0. (0.01 M).

### Fungal and microbial count

The mold prevalence in grains, expressed in colony-forming units per gram (cfu/g), was determined using the methodology described in references^28,29^. Prior to undergoing digestion for 2 min, representative samples weighing 25 g were immersed in a solution of sterile peptone (0.1% concentration in water) for 30 min, with a volume of 250 ml. A volume of 1 mL of the sample underwent a series of dilutions in 9 mL of peptone water. Subsequently, 100 µL of the resulting serial dilution was placed onto dichloron glycerol-18 (DG-18) agar medium (Oxoid chemicals, Hampshire, UK). The agar plates were then incubated at a temperature of 35 °C for a period of 4–5 days. The quantification of colonies (colony-forming units per gramme) was documented subsequent to the incubation period.

### Statistical analysis

A factorial experiment employing a 2 × 4 design, consisting of bag types and storage periods for two cultivars (Nubaria 1 and Giza 716) separately, was conducted using a completely randomized design. The purpose of this experiment was to investigate the impact of bag type, which was manipulated at two levels (3-layer and 7-layer), and the storage period, which was manipulated at four levels (6, 12, 18 and 24 months), on the experimental outcomes. Each treatment combination was replicated three times, resulting in a total of 24 treatments for two cultivars separately. The analysis focused on examining the impact of individual variables on various changes in bean quality parameters, including seed bulk temperature and relative humidity, CO_2_ and O_2_ concentration, moisture content, shearing and penetration force, germination, EC, protein, color change, total phenolic and tannins content, acidity value, and fungal and microbial count as indicated in^30^. Data analysis was conducted using the SPSS 26.0 program (Version 26, IBM Corporation, USA). For multiple comparisons, two-way analysis of variance (ANOVA) and post hoc (Tukey test) were employed. Differences were considered statistically significant at the 0.05 level.

### Research involving plants

The seeds of the two faba bean cultivars (Giza 716 and Nubaria 1) were obtained from the Field Crops Research Institute, Agricultural Research Centre (ARC), Egypt, following the international norms, legislation and guidelines.

## Results and discussions

### Bulk temperature and relative humidity

Table 2 shows the measured seed bulk temperature inside the examined hermetic bags. It was noted that the bulk temperature of seeds stored inside both bags was lower than (30 °C). The results also confirmed that the temperature inside the tested bags fluctuated in a lower range throughout the day than in comparison with the ambient temperature. By ANOVA analysis, the bulk temperature of beans differed significantly throughout the storage months (p < 0:05; HSD_05_ = 5.308 and 5.305 for 3 and 7-layers hermetic bags, respectively). At the same time, there were no significant differences between the bags and the surrounding condition, nor the interaction between the storage period and the types of bags. Grain temperature was always lower than the ambient temperature due to the presence of an anti-UV layer over the surface of the plastic films, which reflects most of the ultraviolet rays that cause the stored grains to rise in temperature, as mentioned in^31^.

**Table 2:** Change in bulk temperature (°C) and relative humidity (%) as related to storage time and inside the two used hermetic bags for both studied faba bean cultivars (Nubaria 1 and Giza 716).

Faba bean variety	Bag type	Temperature (°C) and relative humidity (%)	Storage period (months)			
			6	12	18	24
		Ambient temperature (°C)	16.48 $\pm$ 5.28 b	30.37 $\pm$ 3.06 a	19.10 $\pm$ 6.67 b	30.26 ± 3.79 a
Nubaria 1	3 layers	Bulk temperature (°C)	16.07 $\pm$ 0.25 b	27.63 $\pm$ 0.21 a	17.67 $\pm$ 0.31 b	28.80 $\pm$ 0.30 a
	7 layers		15.70 $\pm$ 0.36 b	26.60 $\pm$ 0.36 a	16.23 $\pm$ 0.31 b	27.37 $\pm$ 0.25 a
Giza 716	3 layers		15.77 $\pm$ 0.21 b	28.80 $\pm$ 0.26 a	19.10 $\pm$ 0.26 b	29.97 $\pm$ 0.32 a
	7 layers		15.37 $\pm$ 0.29 b	26.40 $\pm$ 0.46 a	17.10 $\pm$ 0.20 b	28.87 $\pm$ 0.15 a
		Ambient relative humidity (%)	62.50 $\pm$ 13.20	64.31 $\pm$ 15.40	63.81 $\pm$ 15.11	65.57 $\pm$ 13.84
Nubaria 1	3 layers	Relative humidity (%)	60.60 $\pm$ 0.10	58.93 $\pm$ 0.31	59.90 $\pm$ 0.78	61.00 $\pm$ 0.20
	7 layers		57.17 $\pm$ 0.06	58.60 $\pm$ 0.20	59.40 $\pm$ 0.20	59.77 $\pm$ 0.25
Giza 716	3 layers		57.83 $\pm$ 0.06	58.53 $\pm$ 0.21	58.77 $\pm$ 0.32	59.53 $\pm$ 0.45
	7 layers		55.47 $\pm$ 0.15	56.20 $\pm$ 0.20	56.43 $\pm$ 0.31	57.53 $\pm$ 0.31

*The mean $\pm$standard deviation (SD) was used to express the data; means followed by distinctive lowercase letters indicate significantly different results (P < 0.05) at a 5% significance level.

*The absence of letters indicates the insignificance of differences between treatments.

Cook et al.^32^ indicated that the anti-UV white surface helps reduce the temperature inside the silo, which is one of the essential features of the material used as the outer layer. Meanwhile, outside air temperatures, grain respiration, and insects' presence influence a store's temperature of grains^33^. The decreased temperature inside both studied bags compared to ambient temperature could also be attributed to the lower respiratory rate, thus avoiding early spoilage during storage. In general, the lower temperature of grain bulk inside the stored bags reflected good seed condition and no deterioration of seeds.

Analysis of variance also indicated no significant differences for storage period, bag types, and interaction between them (p > 0:05) on relative humidity inside bags and the surrounding ambient conditions. It may be due to the permeability of water vapor for the two types of plastic films being identical by less than 2, as indicated in Table 1.

The findings show that the ambient relative humidity had no effect on the relative humidity inside the bags. This means that the sealed nature of the hermetic bags prevented any absorption of the surplus moisture from the surrounding atmosphere, which is in agreement with^34^.

Furthermore, a comparison between the seeds stored in the 3-layer bags and the seeds stored in 7-layer bags showed lower relative humidity (RH) inside the bags, which could be due to lower oxygen permeability (≤ 0.1), which slows respiration rate and inhibits the growth of microbes and fungi, both of which would increase the relative humidity around the seeds.

### Carbon dioxide (CO_2_) and oxygen (O_2_) concentrations

**Figure 1:**
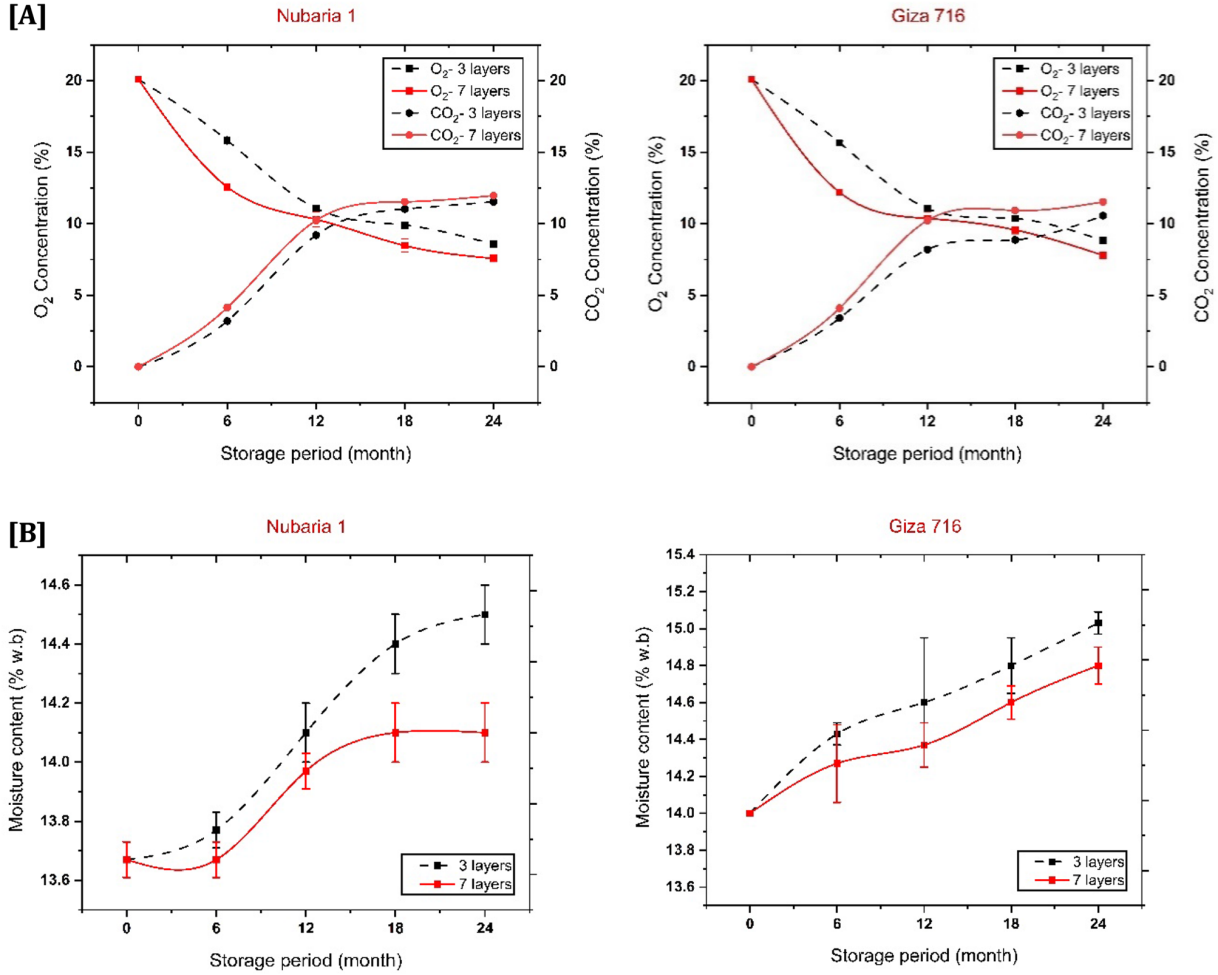
Change in **(A)** CO_2_ and O_2_ concentrations and **(B)** Moisture content (% w.b) among different storage periods (6, 12, 18 and 24 months) as related to types of the hermetic bag (3-layer and 7-layer) and faba bean cultivars (Nubaria 1 and Giza716). *Different lowercase letters on top of the bars indicate significant differences (P ≤ 0.05).

The results in Fig. 1A show the carbon dioxide and oxygen levels during 6, 12, 18, and 24 months of storage. The findings revealed that the average percentage of CO_2_ increased with the progress of storage time. In contrast, the average O_2_ percentage gradually decreased depending on the storage bag type and thickness. For the effect of studied bags, 7 layers resulted in the highest CO_2_% and the lowest O_2_%, which may be attributed to the layers thickness of these bags, which prevents the leakage of gases from it (Oxygen permeability from bag ≤ 2 Cc/m^2^/day for 7-layers). The statistical analysis showed a significant difference (P < 0.05) between the bags and the storage period for both studied cultivars, Nubaria 1 and Giza 716. The CO_2_% significantly increased, and O_2_% decreased in the 7-layer bag compared to the 3-layer one.

It is noted that the difference in carbon dioxide and oxygen percentages inside the hermetic bags might depend upon the seeds' respiration rate and the level of microorganisms inside the bags. Furthermore, it can also be affected by the permeability of the plastic films and its ability to maintain the appropriate gaseous balance during the storage period^35,36^.

The results showed that at the end of the storage time, carbon dioxide levels reached 11.53 and 11.97% for the 3-layer and 7-layer bags with seeds of Nubaria 1. On the other hand, nearly the same range was achieved (10.57 and 11.53%) for Giza 716 (Fig. 1A). These higher levels of CO_2_ reflect the sealing effect of the tested hermetic bags on keeping a favorable gas condition inside the bags.

### Moisture content of the grains

The moisture content of bean seeds is the most critical factor during storage. Seeds with a moisture content greater than 18% risk severe damage during storage and processing. Excessive initial humidity, on the other hand, promotes discoloration, flavor development, loss of water absorption, and mold growth^36^.

It is evident from Fig. 1B that the moisture content of both cultivars' seeds increased slightly for both the 7-layer bags and the 3-layer bags up to 12 months of storage but significantly increased to 24 months of storage. The percentages of moisture content recorded in the 3-layer hermetic bag for both cultivars Nubaria 1 and Giza 716 were 14.50% and 15.03%, respectively, after 24 months of storage Fig. 1B. The rise in moisture content of the seeds might be due to the hygroscopic nature of the seeds and the increase in relative humidity through the monsoons' season^37^. On the other hand, the results illustrated in Fig. 1B showed that the moisture content of stored and relatively increased even under the 3-layer hermetic bag or the 7-layer one. This means that, because of the low permeability of its layers, storage in hermetic bags keeps the moisture content of stored seeds from fluctuating. As shown in Fig. 1B, the 3-layer hermetic bag showed a higher moisture content of seeds than a 7-layer bag for both cultivars. Finally, it may be attributed to their difference in thickness and water vapor permeability, which was recorded in Table 1.

### Shearing and penetration force

**Figure 2:**
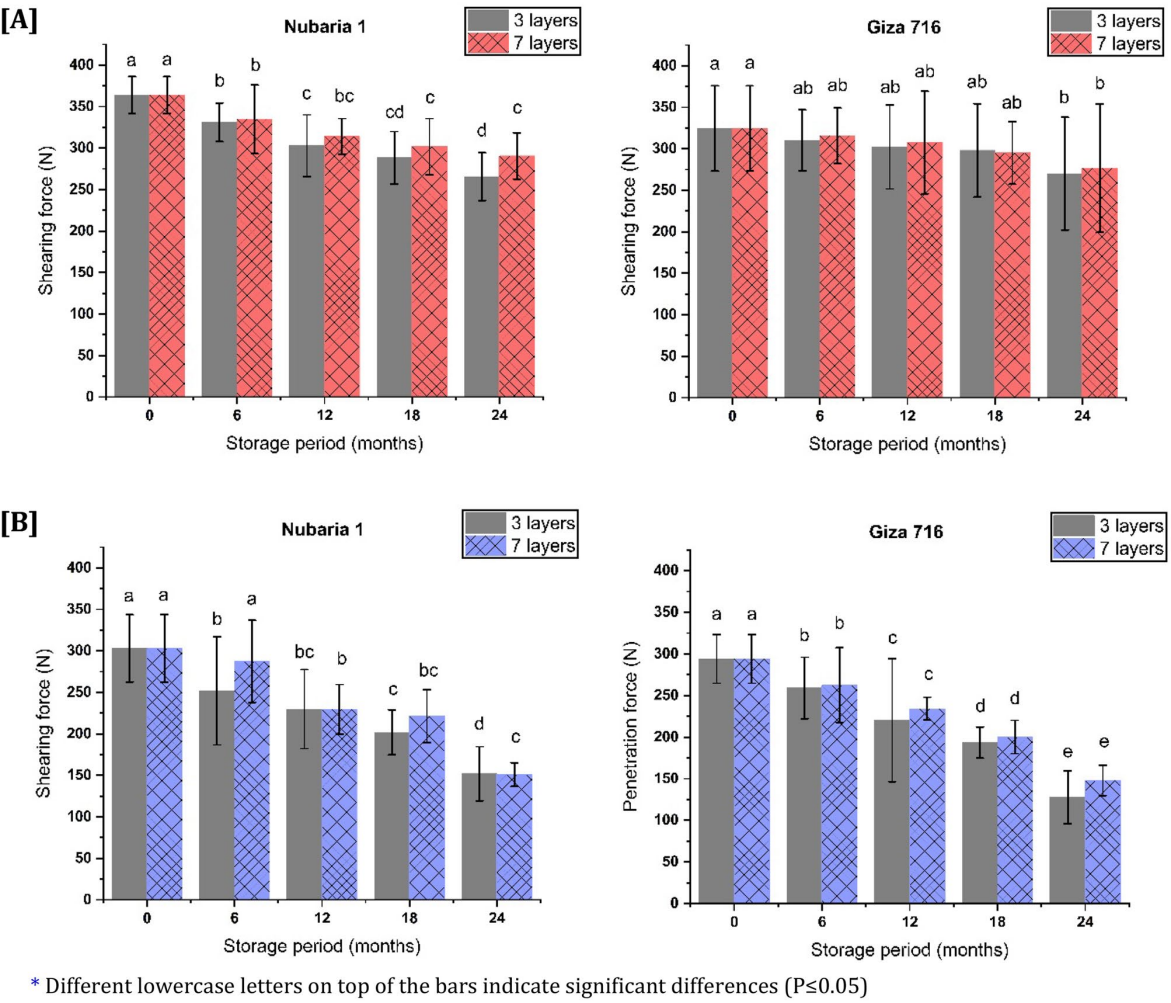
Effect of bag types on **(A)** Shearing force and **(B)** penetration force for two faba bean cultivars (Nubaria1 and Giza 716) after different storage periods (6,12,18, and 24 months).

As shown in Fig. 2A, shearing force is presented for the two types of stored faba beans. The results revealed that the Nubaria 1 sample had a higher initial shearing force than Giza 716, while after 24 months of storage, both cultivars showed lower values. It was observed that the shearing force significantly decreased (P ˂ 0.05) as the storage period increased for both cultivars (Nubaria 1 and Giza 716). For the statistically significant difference between means of storage time, the values of HSD at 5% were 26.22 and 47.78 for Nubaria 1 and Giza 716, respectively.

The average initial kernel penetration force was 294 and 303 N for Giza 716 and Nubaria 1, respectively. However, these values were significantly decreased (P < 0.5, HSD_0.05_ = 16.07 and 35.84) at the end of the storage period for the two bean cultivars. The penetration force of the seed decreased during 24 months of storage to almost 50% at the end of storage for Nubaria 1, while it reached approximately 56% for Giza 716, with no significant differences between the two types of bags, as presented in Fig. 2B.

The current experiment's shear and penetration force results are also consistent with the hardness results of Lee et al.^38^ and El-Kholy and Kamel^28^. The loose structure of starch, which is expected to produce a large area inside the matrix and enhance vulnerability to enzyme attack, could explain the reduced shearing and penetration forces during the storage period^39^. The results may also be due to the slight increase in the bean's moisture content at the end of storage time.

### Germination parameters

**Figure 3:**
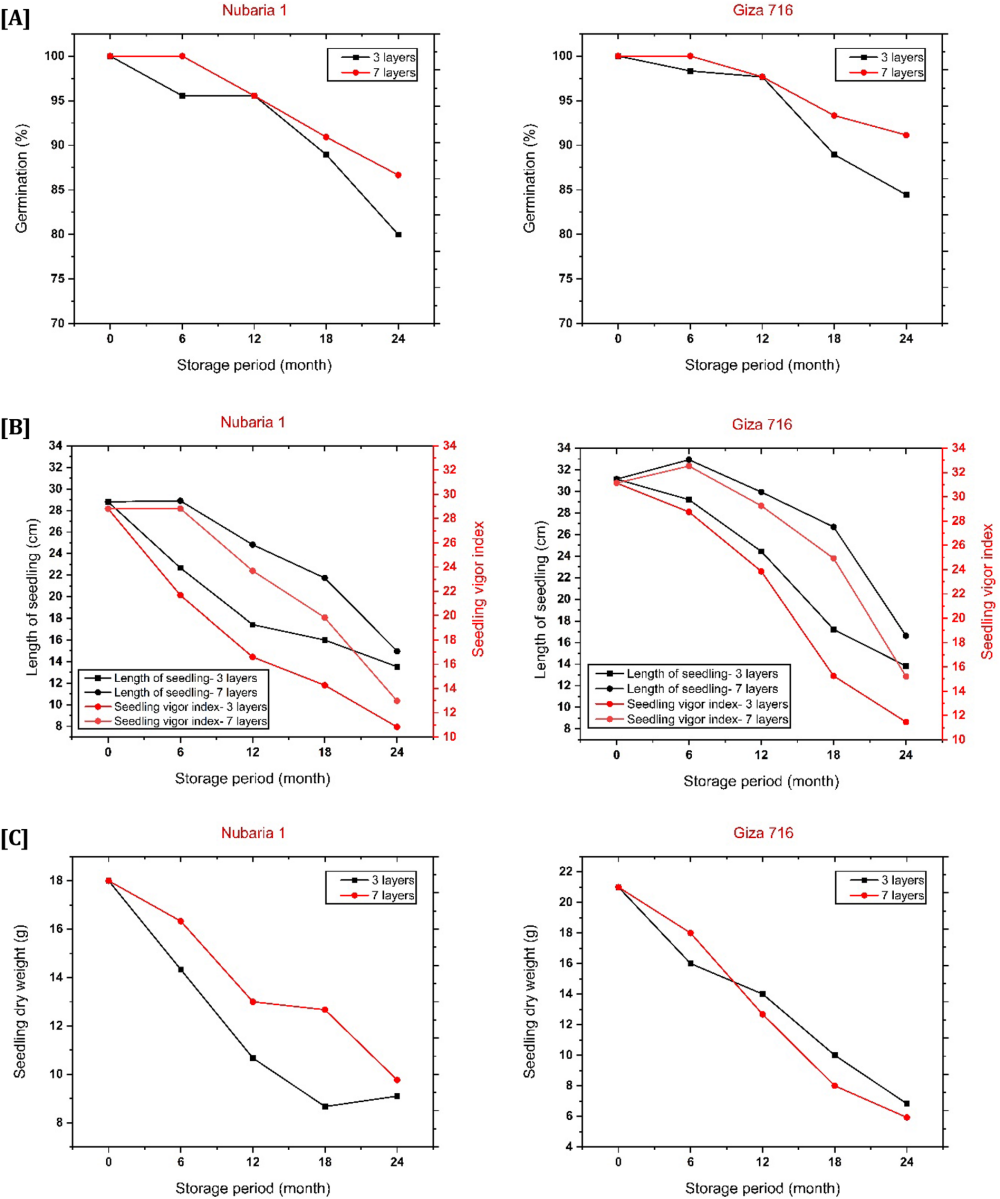
Change in **(A)** germination percentage, **(B)** seedling length and seedling vigor index, and **(C)** dry weight of seedlings during different storage periods related to hermetic bag type (3-layers and 7-layers) and two faba bean cultivars (Nuaria 1 and Giza 716). * Absence of letters on top of the bars indicates insignificant differences in interaction between bag types and storage period (P $\ge$ 0.05).

The germination percentage has been assessed to report if faba bean seeds can be successfully shown and given to a plant in the field after being stored for a long period. Figure 3A demonstrates that for the two cultivars stored in both hermetic bags, the germination % did not considerably decrease (P > 0.05) over the 6 and 12 months of storage. The germination of faba bean seeds significantly declined throughout the 24 months of storage, reaching 80% and 84.44% for the Nubaria 1 and Giza 716 cultivars stored in the 3-layer hermetic bags. However, in the 7-layer hermetic bags, the germination was significantly higher during the same period than in the 3-layer one (86.67% and 91.14%) for the Nubaria 1 and Giza 716 cultivars, respectively. The reduced physiological activity of seeds may be caused by low oxygen tension in the hermetic storage environment^40^. Similarly, results were observed for mung bean grains after six months of storage^41^. Moreover, it can be observed that the germination % is negatively related to the moisture content^18^. In contrast, our results showed that the MC% increased at the end of the storage time for both cultivars at both types of bags Fig. 1B; these bags preserved the germination % by more than 80%. This may be attributed to the fact that the moisture content increase was due to the rainy season at the end of storage, which leads to an increase in the RH% in the outdoors (the condition of storage) and not due to the respiration of the microorganisms. Hence, this increase of MC% did not negatively affect the germination%. Additionally, this was so clear in the increase of MC% of seeds inside 3-layers more than that of 7-layer bags, resulting in a decrease of germination % in 3-layers more than that in 7-layers bags for both cultivars at more than 12 months storage for both cultivars. The results in Fig. 3A illustrate that faba bean seeds recorded high germination % after 6 months of storage and 12 months for the examined 3 and 7-hermetic layer bags. The recorded values for germination (%) were 95.55%, 95.57% at the 3-layer bags and 100%, 95.55% at 7-layer bags, respectively, for Nubaria 1 cultivar and the corresponding values for Giza 716 cultivar were 98.33%, 97.67% and 100%, 97.67%, respectively. The mechanism of keeping the 7-layer hermetic bags to maintain a higher germination percentage during the storage period than that of 3-layer hermetic bags might be attributed to its several layers that protect the stored seeds from the external environment, particularly the temperature, which is a crucial factor that affects the germination^42^, and the inside conditions still stable. The rise in temperature inside these hermetic bags throughout the storage was brought on by the heat produced by the faba bean seeds respiring while it was being stored. The beans exhibited respiration and thermogenesis due to their living state. The temperature of the beans inside both storage systems rose as a result of this heat. Thus, the hermetic bags maintained the germination % of bean cultivars, although the temperature increased at the end of the storage period. Additionally, the change in germination percentage at the end of storage time in the 7-layer hermetic bags might be because of the genetic characteristics of the cultivar itself^2^, which was obvious in Fig. 3A that the Giza 716 cultivar recorded a higher % of germination than Nubaria 1 cultivar 91.14% and 86.67% respectively.

#### Seedling vigor index and seedling length

Figure 3B shows that the seedling vigor index decreased regardless of packaging type as storage time increased for both studied cultivars. As the seeds age increases, the reduction in vigor and viability may be caused by changes in the enzymes that scavenge free radicals, an increase in free radical production, protein degradation, or an increase in the amino acid pool^37^. The seedling vigor is composed of seedling length and germination, so the higher the percentage of germination with longer seedlings, the more the seedling vigor index has been achieved. The cultivar Nubaria 1 is considered a large-sized seed with a seedling vigor index of 28.81 due to its seedling length of 28.81 cm and germination of 100%, as shown in Fig. 3B. The seedling vigor index exhibited considerably higher values for Nubaria 1 stored in the 7-layer hermetic bags (28.90, 24.83, 21.73) than in the 3-layer bags (22.57, 17.43, 16) among the 6, 12 and 18 months of storage, respectively, followed by the minor change between them after 24 months of storage (14.97 and 13.53) for both bags, respectively. These results are interesting to maintain the stored seeds of Nubaria 1 cultivar for 12 months without a marked decline in the seedling length, ascribed to the sufficient nutrients in the seed responsible for feeding the root and shoot to grow and elonged; as a result, to increase the seedling vigor index. Furthermore, large seeds affected seedling growth and development because they contained more energy that could be used to support early seedling growth^43^, which explains the lesser effect of long-period storage in Nubaria 1 seedling length (radical and plumule) than the seedling vigor index of cultivar Giza 716 which has smaller sized Seed Fig. 3B.

#### Seedling dry weight

The dry weight (DW) of both cultivars Nubaria 1 and Giza 716 after 24 months of storage exhibited an excessive decrease for the seeds stored in both types of hermetic bags by around 50%, as shown in Fig. 3C. Concerning Nubaria 1, seeds stored in the 7-layers hermetic bags maintain higher seedling DW than that stored in the 3-layer bags as shown in Fig. 3C among the 6, 12 and 18 months of storage. The superior seedling DW for the seeds stored in the 7-layer bags is attributed to their capacity to reduce storage stress on the seeds and maintain the composition that provides the seedling with nutrients to raise its DW. Nevertheless, after 24 months of storage, there was no significant difference (p > 0.05) between the 7-layer hermetic bags and the 3-layer ones (9.77 g and 9.10 g), respectively. Although there was negligible change in the seedling DW of the Giza 716 cultivar at 24 months of storage (p > 0.05) using the two different types of hermetic bags Fig. 3C, it was less than that of the seedling DW of Nubaria 1 by (3.84 g and 2.27 g) for the 7-layer and the 3-layer hermetic bags, correspondingly.

### Electric conductivity (EC)

**Figure 4:**
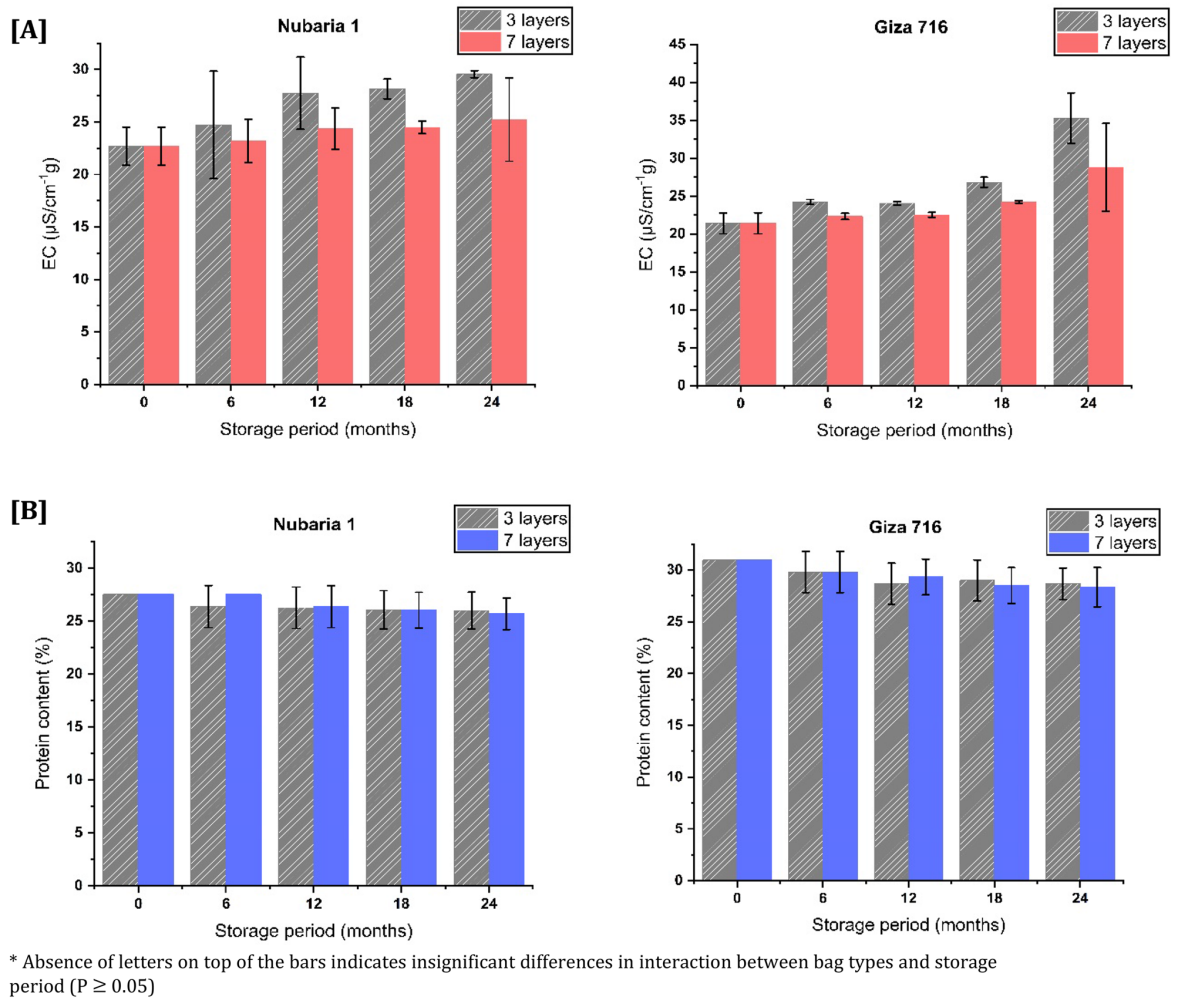
**(A)** Electric conductivity and **(B)** crude protein content of two faba bean cultivars (Nubaria 1 and Giza 716) stored for different periods (6, 12, 18 and 24 months) in (3-layers and 7-layers) hermetic bags.

Electric conductivity (EC) measures the number of ions released from the seeds to the hydration water^44^. These ions are created when phytic acid in the bean breaks down (mostly divalent cations). Electric conductivity is influenced by several variables, including initial moisture content, physical weaknesses, and storage conditions^45,46^. The result of EC that is illustrated in Fig. 4A shows that for both cultivars, seed storage in the 7-layer hermetic bags recorded lower EC values than that stored in the 3-layer hermetic bags all over the storage periods except at the first 6 months of storage; there was no significant difference (P > 0.05) between the two bags for cultivar Nubaria 1 and at the 12-months of storage for cultivar Giza 716. Furthermore, it is obvious in Fig. 4A that EC significantly increased at the end of the storage period for cultivar Giza716 (352.7 $\pm$ 3.34) compared to the initial storage time of seeds (214.0 $\pm$ 1.38) in the 3-layer hermetic bags, which is due to the increase of moisture content of those seeds. The upsurge in moisture content may lead to fungus, which deteriorates the cell membrane and increases cell disruption^47^. Accordingly, the more faba beans cell disturbance, the higher the electric conductivity occurred of seeds.

### Crude protein content

Faba beans have a protein content ranging from 18 to 32%, depending on the genotype^48^. Figure 4B shows that cultivar Nubaria 1 contains (27.52%) protein content which is less than that of cultivar Giza 716 (30.96%). At the end of the storage period, the protein content decreased in both cultivars (P > 0.05), with superior content for Giza 716 in the two types of hermetic bags, as investigated in Fig. 4B. These results are in harmony with^49^. It may be that proteolytic enzymes are responsible for the gradual decline of crude protein content in faba bean seeds.

### Color change

During harvest, the most common color of the seed coat (testa) on a faba bean is a light brown or beige hue; however, this hue is inconsistent and becomes darker after being stored^50^. Conditions during storage significantly affect how long the color of the bean seeds will remain after storage. Previous research indicates that temperature, seed moisture content, and relative humidity are the primary factors influencing seed color stability in legumes during storage^51,52^, so there are significant changes in the color parameters of stored beans.

**Figure 5:**
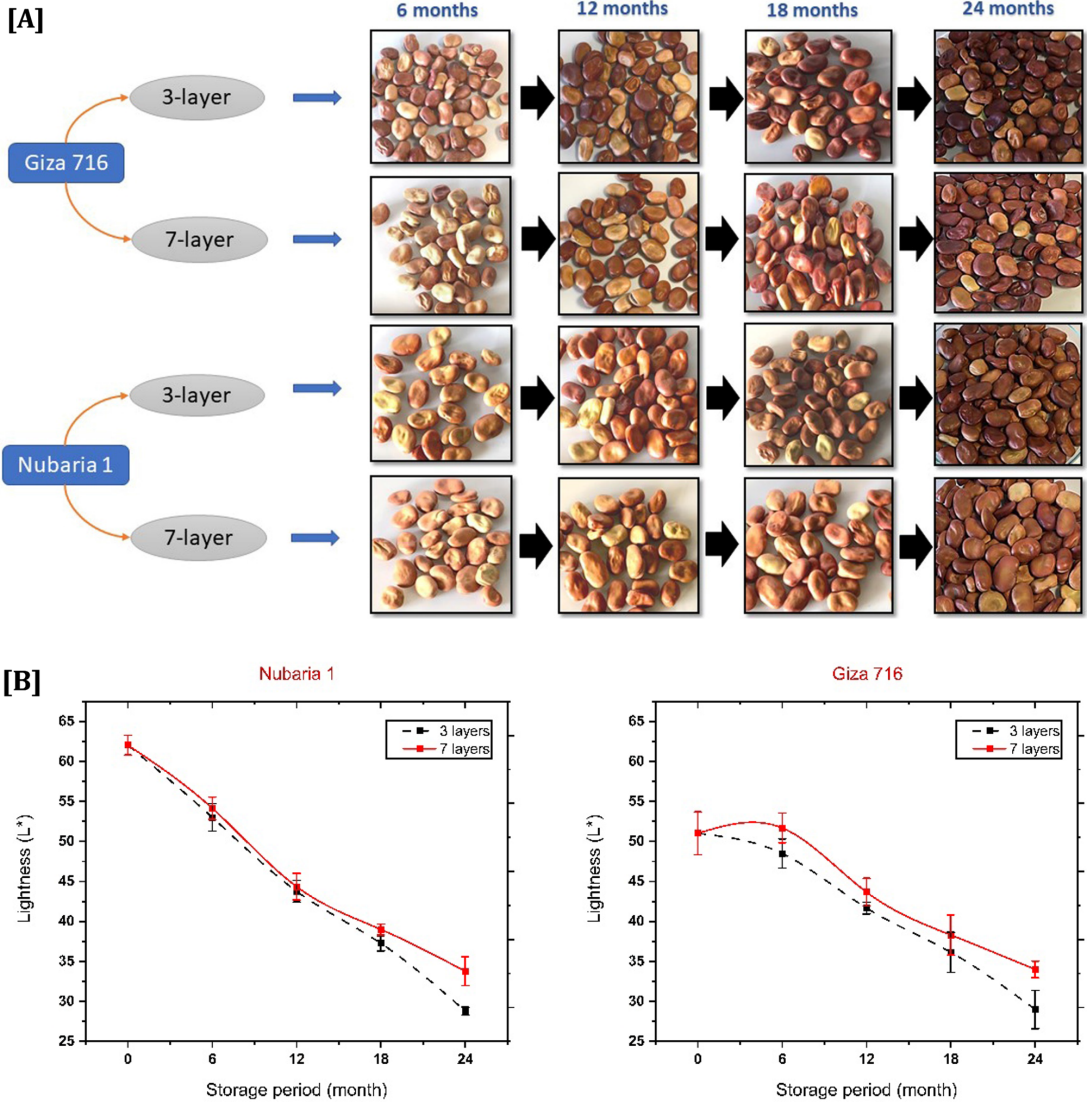

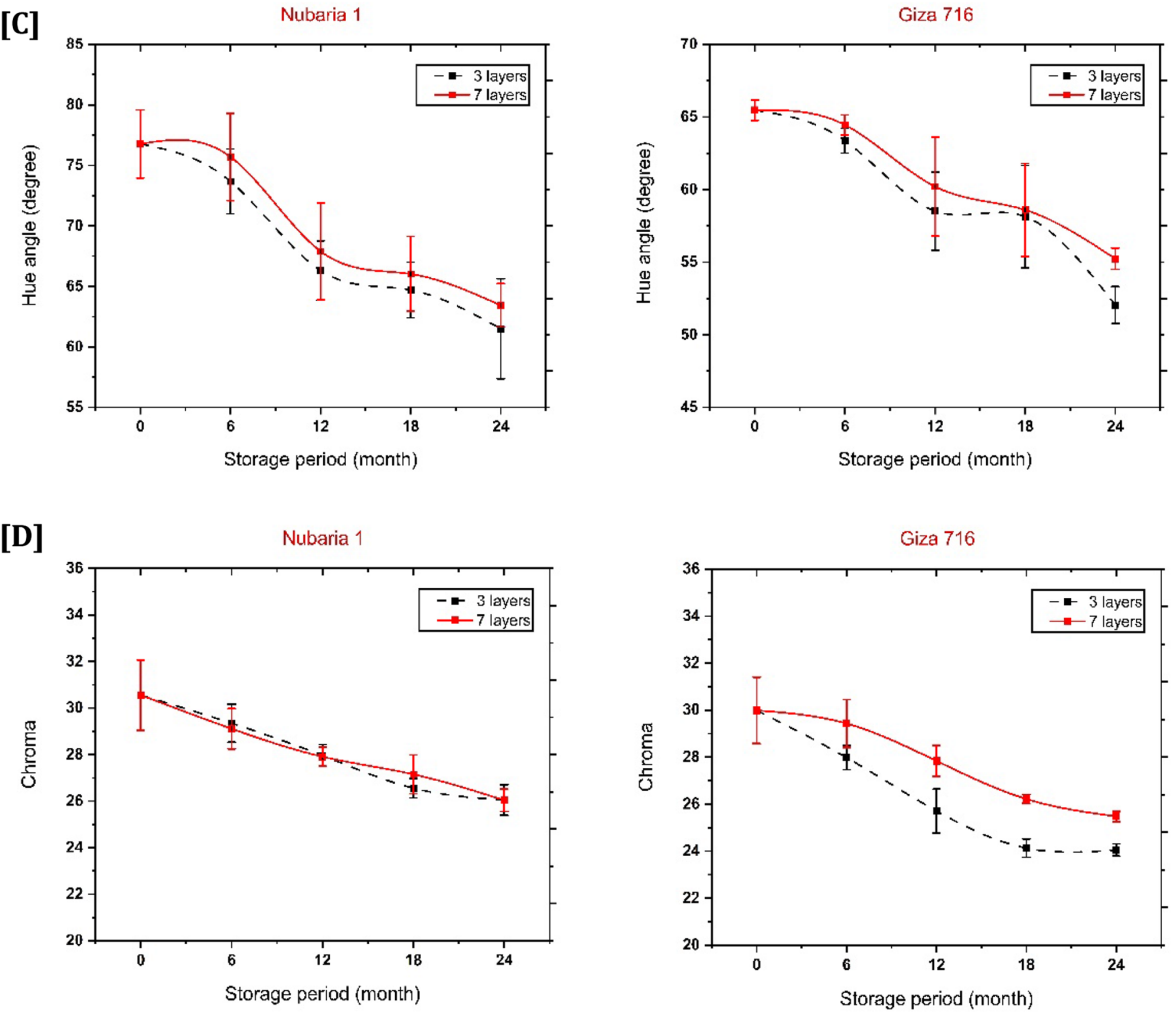
Effect of bag types (3-layers and 7-layers) hermetic bags on (**A**) the color testa, (**B**) lightness, (**C**) chroma, and (**D**) hue angle for two faba bean cultivars (Nubaria 1 and Giza 716) among various periods of storage (6, 12, 18 and 24 months).

The type of bags, cultivars and storage duration influenced faba bean testa color, as shown in Fig. 5A. On the other hand, Fig. 5B–D describes the effect of the three color coefficients, where luminosity indicates lightness or darkness (100 for complete white and 0 for complete dark). Chroma represents the intensity of the color; the higher the value, the greater the intensity. The hue angle represents various spectrum colors on a 360° axis, classifying the colors of red, yellow, blue, etc. As demonstrated in Fig. 5B, the findings of brightness fell significantly and became darker after 12 months (P0.05, HSD_0.05_ = 1.54 and 3.74 for Nubaria 1 and Giza 716, respectively). Seeds were much darker at the end of storage and fell to 28.99 and 34.40, with a loss of 43.16 and 32.56 for Giza 716 held in 3-layer and 7-layer bags, respectively. At the same time, the lightness value was 28.78 and 33.77 for Nubaria, with loss percentages of 53.60% and 45.56%, respectively. The findings indicated that an acceleration in respiration rate, moisture content, and enzymatic processes led to browning reactions in the darker samples at the end of the storage period^46^. It was also found that beans at moisture levels (more than 14.5%) significantly increased the color deterioration during storage, which occurred after a storage period of 12 months, but storage in 7-layer bags was always better than in 3-layer bags.

The changes in chroma of Giza 716 seeds with the storage period were slight (P < 0.05, HSD_0.05_ = 2.40) and decreased the intensity of the color by 19.80% and 18.37 for the 3-layer and 7-layer bags, respectively. However, no significant interaction differences were relative to Nibaria 1 (P > 0.05). Still, differences were significant between storage months (P < 0.05, HSD_0.05_ = 1.53), Fig. 5C. High oxygen levels during long-term storage accelerate the darkening intensity of faba bean color^50^. In contrast, in the present investigation, low oxygen levels slowed the change in color intensity (Chroma). During storage, faba beans may lose their vibrant color due to the oxidation of their leucoanthocyanidins catalyzed by air and light^51^.

Results revealed that the hue angle was decreased for Giza 716 (P < 0.05, HSD_0.05_ = 3.98) without a significant difference for Nubaria 1 (P > 0.05), as shown in Fig. 5D. The hue angle changed from 76.77 to 61.50 and 63.43 with different storage bags (3-layers and 7-layers, respectively) for Nubaria 1. However, the reduction in hue angle of Giza 716 ranged from 64.46 to 52.05 and 55.24 for the 3-layer and 7-layer bag storage, respectively. These results are similar to those encountered by Nasar-Abbas *et* al.^14^ and Siqueira et al*.*^53^, who confirmed the reduction of hue angle at the end of the storage period of faba beans.

### Total phenolic content (TPC)

Table 3 exhibits no effect for the type of bags on the TPC in seeds of Nubaria 1 (P > 0.05) and conversely for cultivar Giza 716. The 7-layer hermetic bags markedly affected the seeds of Giza 716 by generating more TPC than the 3-layer bags (29.80, 28.29, 20.54 mg/g and 26.89, 25.34, 19.27 mg/g) during the periods of storage 6, 12 and 24 months, respectively. For the prolonged storage time, the TPC in cultivar Nubaria 1 recorded a slight increase among the periods of storage in the 3-layer hermetic bags and the 7-layer bags until the end of 18 months (24.32 mg/g and 27.23 mg/g), respectively. Nevertheless, the TPC decreased significantly by ≈ 41.9% and 28.9% at the end of 24 months of storage in both the 3 and 7-layer hermetic bags, respectively Table 3, which led to an increase in the darkness of testa color of cultivar Nubaria 1. In addition, Cultivar Giza 716 exhibited the same trend as Nubaria 1; nonetheless, the decline of TPC after 24 months compared with 18 months of storage was (≈21.8% and ≈14.9%) in the 3 and 7-layer bags, respectively. The oxidative degradation of specific phenolic compounds may cause a drop in phenolic substances as color darkening increases. Moreover, storing numerous cultivars of faba beans under low oxygen concentrations inhibits color darkening, indicating that the oxidation of polyphenolics is the cause of decreasing the phenolics, followed by color darkening^29,47^. In particular, non-tannin polyphenols may be mainly susceptible to oxidation due to peroxidase enzyme activity that persists during postharvest conditions for storage^54^, which explains the results of our experiment was a decrease of TPC in the 7-layer bags, which prevented much light from penetrating the bag due to the inner black layer of the bags so no more light exposure for the seeds with less testa darkening for both cultivars.

**Table 3:** Change in the total phenolic content (mg/g), total tannin content (μg/g), and the acidity value (%) of the two faba bean cultivars (Nubaria 1 and Giza 716) as related to different storage periods (6,12,18 and 24 months) in 3-layers and 7-layers hermetic bags.

	Faba bean variety	Bag type	Storage period (months)				
			0	6	12	18	24
Total Phenolic content (mg/g)	Nubaria 1	3 layers	19.72 $\pm$ 7.00 ab	22.13 $\pm$ 1.58 a	22.37 $\pm$ 2.07 a	24.32 $\pm$ 2.10 a	14.12 $\pm$ 1.03 b
		7 layers	19.72 $\pm$ 7.00 b	21.57 $\pm$ 4.38 ab	21.56 $\pm$ 1.25 ab	27.23 $\pm$ 0.86 a	19.36 $\pm$ 1.33 b
	Giza 716	3 layers	24.38 $\pm$ 0.52 a	26.89 $\pm$ 0.92 b	25.34 $\pm$ 2.14 b	24.66 $\pm$ 0.33 a	19.27 $\pm$ 1.64 b
		7 layers	24.38 $\pm$ 0.52 a	29.80 $\pm$ 0.36 a	28.29 $\pm$ 2.10 a	24.15 $\pm$ 0.82 a	20.54 $\pm$ 0.74 a
Total tannin content (μg/g)	Nubaria 1	3 layers	2.67 $\pm$ 0.29 ab	2.58 $\pm$ 0.52 ab	2.33 $\pm$ 0.36 b	2.25 $\pm$ 0.25 b	2.00 $\pm$ 0.00 b
		7 layers	2.67 $\pm$ 0.29 ab	3.00 $\pm$ 0.25 ab	3.42 $\pm$ 0.29 ab	3.50 $\pm$ 0.25 a	3.48 $\pm$ 0.46 a
	Giza 716	3 layers	0.96 $\pm$ 0.26 b	1.67 $\pm$ 0.14 b	2.25 $\pm$ 0.25 ab	2.50 $\pm$ 0.25 a	1.25 $\pm$ 0.25 b
		7 layers	0.96 $\pm$ 0.26 c	1.92 $\pm$ 0.76 b	2.50 $\pm$ 0.66 ab	2.83 $\pm$ 0.63 a	1.33 $\pm$ 0.14 bc
Acidity (%)	Nubaria 1	3 layers	7.13 $\pm$ 0.15 c	9.62 $\pm$ 0.11 c	13.50 $\pm$ 2.49 b	16.80 $\pm$ 0.86 ab	18.71 $\pm$ 0.90 a
		7 layers	7.13 $\pm$ 0.15 c	9.20 $\pm$ 0.17 c	15.42 $\pm$ 0.59 b	16.12 $\pm$ 0.10 ab	16.90 $\pm$ 0.85 ab
	Giza 716	3 layers	8.07 $\pm$ 0.06 e	9.20 $\pm$ 0.37 e	13.82 $\pm$ 0.03 c	15.29 $\pm$ 0.36 b	19.41 $\pm$ 0.37 a
		7 layers	8.07 $\pm$ 0.06 e	8.63 $\pm$ 0.81 e	10.86 $\pm$ 1.39 d	14.55 $\pm$ 0.56 bc	15.21 $\pm$ 0.18 bc

*The mean and standard deviation (SD) were used to express the data; means followed by distinctive lowercase letters indicate significantly different results (P < 0.05) at a 5% significance level.

### Total Tannin content (TTC)

Tannins are polyphenolic chemicals that are water soluble and have molecular weights between 0.5 and 3 kDa. They can precipitate proteins in aqueous solutions. Condensed tannins are most frequently found in dicotyledonous plants such as faba bean^55^. Although the deficit in tannin content might be due to the intense antioxidant activity, tannins are 15–30 times more operative than simple phenolics at squelching peroxyl radicals^56^′^57^. Most of the condensed tannins are found in the seed coats, and removing the seed coats reduces the amount of these condensed tannins^58^. Concerning our results of Total Tannins content TTC in Table 3, Nubaria 1cultivar exhibited no significant difference among the storage periods 6, 12, 18, and 24 months (P > 0.05). In addition, the 7-layer hermetic bags caused the simulation of generating more TTC in Nubaria 1seeds by (≈39.1% and ≈42.5%) after 18 months and 24 months of storage than those packed in the 3-layer hermetic bags. The results of Nubaria 1 seeds in Table 3 elucidate this cultivar's ability to maintain its seed's antioxidant activity, alleviating the darkness of its testa and preserving its quality during storage. On the other hand, Giza 716 cultivar revealed a gradual increase in TTC by increasing the storage period in both types of bags until 18 months of storage and reached the highest values (2.50 µg/g and 2.83µ/g) in the 3-layers and 7-layer bags, respectively. While there was a marked increase in TTC in cultivar Giza 716 seeds at 18 months of storage, extending the storage period to 24 months inhibited the production of TTC by (50% and ≈33%) in the 3-layers and 7-layers hermetic bags, respectively Table 3. The superiority of 7-layer hermetic bags to the 3-layers one resulted in protecting the seeds of both cultivars against oxidation and darkening their testa colour, which is an undesired characteristic of faba bean seeds.

### Acidity value

The results in Table 3 illustrate that for cultivar Nubaria 1, there was no significant difference in the acid value after 6-months of storage in both types of hermetic bags (9.62% and 9.20%) in the 3-layer and 7-layer bags, respectively. Moreover, cultivar Giza 716 exhibited the same trend after the same period. In contrast, starting from 12 months of storage till the end of the storage period, the acidity values surged substantially to be (18.7%, 16.90%, and 19.41%, 15.21%) for both cultivars Nubaria 1 and Giza 716 stored in both types of hermetic bags, the 3-layers and 7-layers one, respectively. Similar results for stored seeds in hermetic bags were reported by Liu et al*.*^59^, Afoakwa et al*.*^60^, and Hashempour‐Baltork et al*.*^61^. According to these findings, less acidity could be produced when the liquid evaporation rate from the testa is balanced with the rate of its dispersion from the cell membrane. As a result, prolonged storage will weaken the testa and make it more permeable to the liquid that carries acetic acid to the membrane, allowing the water containing the acids to escape without trapping the acid molecules, similarly in cocoa beans^62^. Furthermore, the efficacy of 7-layer hermetic bags in the preservation of faba bean seeds by delaying the deterioration of seed cells since it achieved low acidity values for both cultivars compared with the 3-layer bag after long time storage (24 months) (16.90% and 18.71%) for Nubari 1 cultivar and (15.21% and 19.41%) for Giza 716 cultivar, respectively.

### Fungal and bacterial count

The results indicate that all stored seeds' combined fungal and bacterial counts after 24 months were approximately 0.67 × 10^2^ and 1.45 × 10^2^ cfu/g, respectively. In the interim, the fresh seeds' initial fungal and bacterial counts, prior to storage, were around 0.79 × 10^2^ and 1.89 × 10^2^ cfu/g, respectively. Notably, no significant variations were seen between the 0 and 24-month storage periods for the seeds. Based on the data provided, it can be observed that the microbiological load of all the seeds tested was found to be lower than the microbiological load limits specified by the Egyptian Specification Standards (ES: 1930/2008 and ES: 2095/2005), as well as the International Standards Organisation (ISO: 9301/2003 and ISO: 2255/1996). This is due to the microbiological specifications outlined in the seeds legislation, which establish maximum limitations of 10^2^ and 10^4^ colony-forming units (CFU) per gram for total fungal and bacterial counts, respectively^28,29^.

## Conclusion

This study showed that the examined multiple layers of hermetic bags could preserve the quality of faba bean seeds stored outdoors for up to 24 months at varying relative humidity and temperatures. In addition, the multiple-layer hermetic bags keep the seeds healthy against microorganisms and their respiratory level stable enough. The 7-layer hermetic bag showed higher seed protection and final quality for faba bean cultivars, which was obvious in maintaining the seeds' viability for long-term storage. Hence, it is recommended for long-term safe storage of faba bean seeds. Farmers who cultivate faba bean seeds can use the technology to store their seeds and benefit from their effective and affordable solution to not only preserve the quality of faba bean seeds with high protein content and nutrition value but also save the place for building stores and improve crop productivity that multiple layers hermetic bags may be used to protect the quality of seeds.

## Data Availability

The data sets used and/or analyzed during the current study are available to readers as in the manuscript and from the corresponding author upon reasonable request.
